# Vasculitis, an Early Unusual Presentation of Rheumatoid Arthritis: A Case Report

**DOI:** 10.7759/cureus.52845

**Published:** 2024-01-24

**Authors:** Ashutosh Kashyap, Biraj Pokhrel, Aaraju Bhatta, Savita Aryal, Shambhu Khanal

**Affiliations:** 1 Department of Internal Medicine, Tribhuvan University Institute of Medicine, Kathmandu, NPL; 2 Department of Internal Medicine, Patan Academy of Health Sciences, Kathmandu, NPL; 3 Department of Emergency Medicine, Lumbini Provincial Hospital, Kathmandu, NPL; 4 Department of Internal Medicine, Lumbini Provincial Hospital, Kathmandu, NPL

**Keywords:** vasculitis, rheumatoid vasculitis, rheumatoid arthritis, gangrene, corticosteroids

## Abstract

Rheumatoid arthritis (RA) is a chronic inflammatory disease, with rheumatoid vasculitis (RV) being its most threatening complication. We report a case of a 70-year-old female presenting with gangrene of the tips of fingers and toes early in the course of RA, which is a rare manifestation. The skin is the most commonly affected organ in RV, followed by the peripheral nerves. However, almost every organ system can get implicated. The management of RV is mostly empirical, with high-dose glucocorticoids and cyclophosphamide. Early diagnosis and optimum management are essential in preventing severe complications of the disease.

## Introduction

Rheumatoid arthritis (RA) is a chronic autoimmune inflammatory disease characterized by inflammation of the synovial joints, primarily the small joints of the hands and feet. As a systemic disease, it has the potential to affect almost all organs and systems of the body, giving rise to numerous extra-articular manifestations. These manifestations have been found to be associated with high titers of rheumatoid factor (RF) and anti-cyclic citrullinated peptide (anti-CCP) [1,2]. Rheumatoid vasculitis (RV) is a rare and one of the most threatening extra-articular manifestations of RA, leading to several cutaneous as well as systemic features. Its annual incidence is found to be below 1% [3]. It is characterized by tissue damage or ischemia and is pathologically correlated with mononuclear or neutrophilic infiltration of the vessel wall, leading to vasculitis and primarily afflicting small- to medium-sized vessels [4].

Despite being rare, it can be a catastrophic condition for patients due to the high rates of morbidity and mortality. Such high rates are associated with both the condition itself and the consequences of the treatment employed. It has been found that the mortality rate within five years of the development of RV can be as high as 40% [5]. However, its incidence has considerably decreased in recent times, probably owing to the introduction of biological disease-modifying anti-rheumatic drugs (DMARDs) [3]. RV is more common in males and is usually seen to occur in patients with long-standing disease [4]. It generally occurs after over 10 years in the clinical course of RA in a patient but has the potential to occur at any stage of the disease [6].

Here, we report a case of a 70-year-old female presenting with gangrenous tips of fingers and toes within six months of the first symptoms of RA who was subsequently diagnosed with RV. Such an early presentation of RV, that too in a female, is quite unusual. Even more perplexing is the fact that this was her first presentation to a healthcare facility.

This case report has been reported in line with the Consensus-based Clinical Case Reporting (CARE) Guidelines [7].

## Case presentation

**Figure 1:**
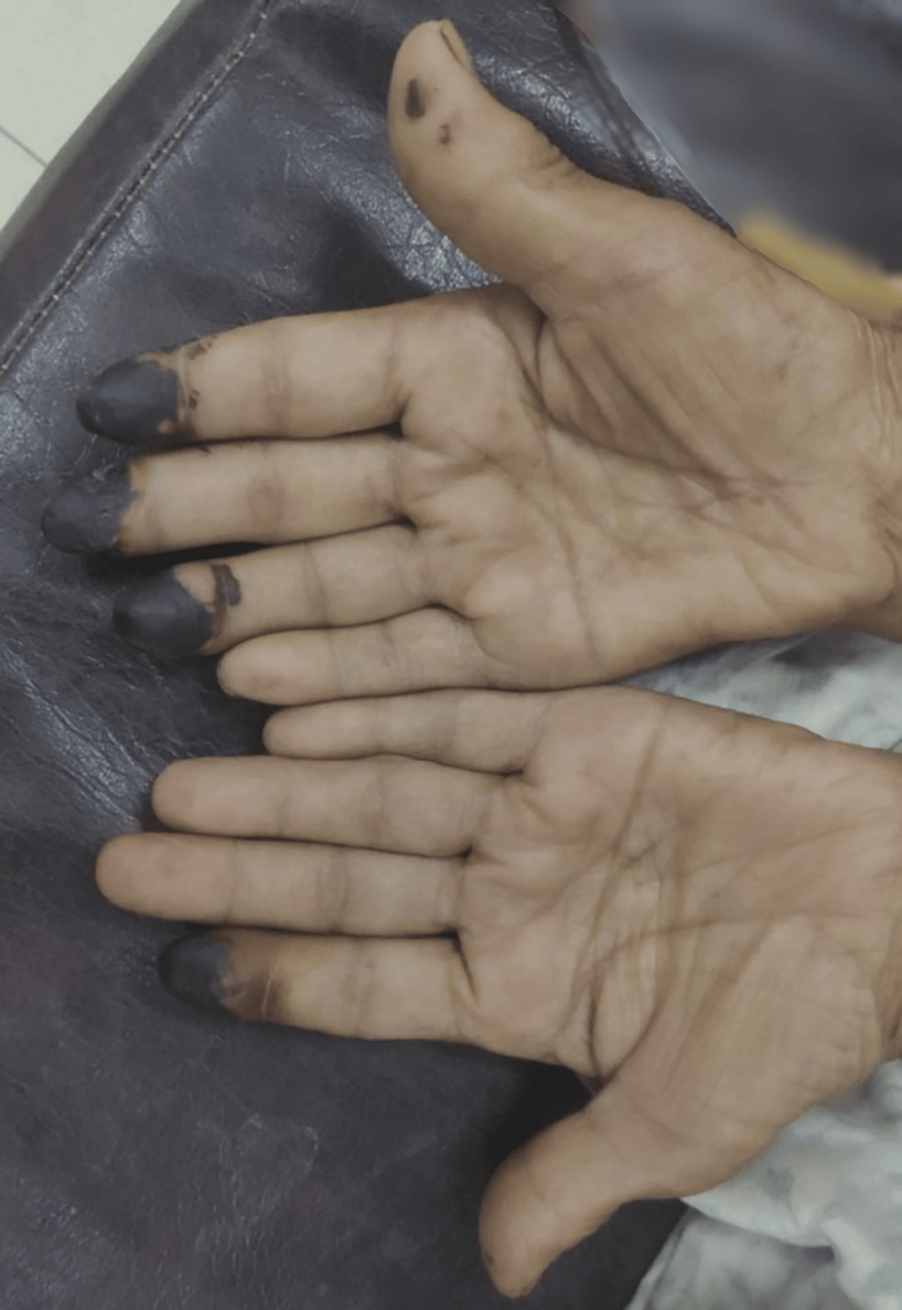
Dry gangrene in both hands

A 70-year-old lady presented to the internal medicine OPD for the first time with blackish discoloration of the tips of her fingers and toes, which started 10 days prior to presentation (Figure 1).

The discoloration was extremely painful and progressive. She was a non-smoker and did not consume alcohol either. There was no history of leg pain or claudication in the past. Upon probing further, she revealed that she had been experiencing pain in multiple joints, including the metacarpophalangeal joints and proximal interphalangeal joints, along with morning stiffness, for the past six months. She had no history of epistaxis, hemoptysis, tingling, or burning sensations of the limbs. She did not complain of dryness in her mouth or eyes. On examination, her general condition was fair, and her vitals were stable, with a blood pressure of 120/80 mm Hg and a pulse rate of 80 bpm. All her peripheral pulses were palpable. There was no pallor, icterus, clubbing, lymphadenopathy, or cyanosis. All findings from the systemic examination were within normal limits.

On investigation, her complete blood counts, renal and liver function tests, serological tests for infections, and urine routine findings were within normal limits. The extractable nuclear antigen panel was negative. Both the cytoplasmic antineutrophil cytoplasmic autoantibody and the perinuclear antineutrophil cytoplasmic antibody were negative. The RA factor was also negative, but anti-CCP came out positive, with titers of >500 units/liter (U/l; reference range: <17 U/l). CRP titers also came back high at 105 U/l (reference range: 0-10 U/l). The findings of peripheral arterial Doppler studies were normal. With these findings, we started her management with a diagnosis of RV. She was started on pulse doses of methylprednisolone (1 g/day for three days, followed by prednisolone 1 mg/kg body weight gradually tapered over three months) and cyclophosphamide (15 mg/kg body weight every two weeks for three doses, followed by maintenance pulses of 15 mg/kg intravenously every three weeks until remission), along with cotrimoxazole (sulfamethoxazole and trimethoprim) prophylaxis. The proximal progression of gangrene halted with treatment; however, we had to amputate her gangrenous digits within two weeks of admission. Due to financial issues, the patient could not afford a biopsy. The patient was better on subsequent visits and had improvements in her joint symptoms as well. She has been kept on long-term OPD follow-up since then.

## Discussion

The pathogenesis behind the development of RV is not completely understood [8]. The deposition of circulating immune complexes containing RF and autoantibodies like anti-endothelial cell antibodies, triggering an inflammatory response with subsequent endothelial cell damage, has been proposed as a possible mechanism [9]. Male gender, smoking, long-standing disease, severe RA, coexistent peripheral vascular disease, cerebrovascular disease, seropositivity for RF, and anti-CCP are considered important risk factors behind the development of RV [4,8]. Although male gender and long-standing disease are more often associated with this condition, our patient developed RV within six months of the onset of symptoms of RA, making it an unusual presentation. Smoking has been demonstrated as the most consistent risk factor, especially in a male seropositive patient [10]. However, the exact trigger behind the development of vasculitis at a particular point of the disease is not known; infections and vaccinations have been cited as possible triggers [6]. We were unable to identify any such triggers that would have kicked off the vasculitis process in our patient.

The skin is the most commonly affected, followed by the peripheral nervous system. However, any organ system can be involved [8]. The cutaneous manifestations include digital infarcts, including nailbed or nail folds, non-healing ulcers, palpable purpura, non-specific maculopapular or nodular erythema, hemorrhagic blisters, livedo reticularis, or digital gangrene [3,8]. Digital gangrene, nail fold infarcts, and large cutaneous ulcers are considered severe manifestations [5]. The peripheral nerves can be involved in the form of progressive sensory or mixed motor and sensory neuropathy, manifesting initially with numbness and gradually progressing to tingling and muscle weakness [6].

Apart from this, RV can affect the eyes in the form of scleritis, episcleritis, or peripheral ulcerative keratitis. It can cause myocarditis, pericarditis, or myocardial infarction in the heart. The bowel and kidneys are less commonly involved. Cerebral vasculitis can present with devastating consequences in the form of ischemic stroke, hemorrhages, seizures, cranial nerve palsies, meningitis, and encephalopathy [11]. In some instances, patients can present with ischemia and infarction in multiple organs in the form of malignant RV. Along with these specific presentations, patients can also have constitutional symptoms such as fever, malaise, fatigue, and weight loss. Elevated levels of acute phase reactants like ESR and CRP, low levels of complements, elevated alkaline phosphatase, and leukocytosis are also associated [10,11].

Before making a definitive diagnosis of RV, it is important to rule out other possible causes behind vasculitis processes. The diagnosis of vasculitis is histopathological; however, it is not always possible, and reliance on clinical presentation and other modalities of investigation becomes significant [5]. Laboratory tests, including high titers of RF and anti-CCP antibodies, are only supportive of a diagnosis of RV and not confirmatory. Although it is a rare manifestation of the disease, early diagnosis and optimum management are of paramount importance as they can curtail untoward complications and improve the overall prognosis of the patient [12]. Due to a lack of data from randomized controlled trials, the management of RV is largely empirical. High-dose glucocorticoids in combination with cyclophosphamide are often used, and sustained treatment with DMARDs is recommended [6,8]. Rituximab, azathioprine, and mycophenolate mofetil are some other agents that are used in its management. Tocilizumab and abatacept have been tried in refractory cases [8].

## Conclusions

RV is a rare but potentially dangerous extra-articular manifestation of RA. Although cutaneous and peripheral nerve manifestations are most common, they can affect virtually any organ system and present with life-threatening complications. As such, a high index of suspicion is necessary with optimal, timely interventions for positive outcomes.
